# Measuring alcohol-based hand rub volume used by healthcare workers in practice

**DOI:** 10.1186/2047-2994-4-S1-P295

**Published:** 2015-06-16

**Authors:** RA Leslie, CJ Donskey, TF Zabarsky, AE Parker, DR Macinga, O Assadian

**Affiliations:** 1 Research and Development, GOJO Industries, USA; 2 Case Western Reserve School of Medicine, USA; 3 Cleveland Veterans Affairs Medical Center, USA; 4 Center for Biofilm Engineering, Montana State University, USA; 5 University of Huddersfield, UK

## Introduction

Recent hand hygiene (HH) research has focused largely on adherence to defined HH indications specified in global and national guidelines. Research focused on quality of HH events has been limited. A critical variable impacting the quality of hand disinfection is the volume of alcohol-based hand rub (ABHR) applied during a single HH event (i.e. application volume or AV). It may be assumed that healthcare workers (HCWs) use AVs recommended by manufacturers; however, HCWs typically are able to control AV. Manufacturer recommendations for AV of ABHR vary based on local requirements with typical recommendations varying from 1 mL to 5 mL. To date, little data exists on actual ABHR AVs used in clinical practice.

## Objectives

Determine the mean AVs of an ABHR used by HCWs during routine clinical practice.

## Methods

Personal carriage bottles (125 mL) with lids engineered to count each ABHR use were used by 19 HCWs from five wards over three shifts in an acute care hospital. The bottles contained the WHO ethanol formulation. The mass of each bottle was measured before and after each shift and the number of uses was recorded after each shift. The total volume of ABHR used by each HCW during a shift was calculated by dividing the total mass used by the density of the product (0.8607 g/mL). The mean AV was determined by dividing the total volume by the number of ABHR uses recorded. Data was analyzed by ANOVA with nested random effects.

## Results

The mean AV of ABHR used by each HCW per shift ranged from 0.27 mL to 1.61 mL per use. The overall mean AV was 0.73 mL (±0.364 SD). The mean AVs were statistically significantly different among the five wards that participated (*p* = 0.0118). The number of HH events from personal carriage bottle per HCW over a shift ranged from 2 to 78 with a mean number of 22 (median 18).

## Conclusion

The results of this study suggest that ABHR AVs used by HCWs are lower than manufacturer recommended volumes. Such practices may have a negative impact on ABHR effectiveness in clinical practice. More extensive studies are needed to understand whether the tendency to use AVs below recommended volumes is widespread, the factors influencing volume preference, and the impact of such practices on antimicrobial product effectiveness.

## Disclosure of interest

None declared.

